# Effect of Intracameral Ophthalmic Cefuroxime Solution (Aprokam^®^) in the Prophylaxis of Cataract Surgery in Patients with Keratoplasty

**DOI:** 10.4274/balkanmedj.2017.1493

**Published:** 2018-03-15

**Authors:** Mustafa Erdoğan Cicik, Cezmi Doğan, Osman Şevki Arslan

**Affiliations:** 1 Department of Ophthalmology, İstanbul University Cerrahpaşa School of Medicine, İstanbul, Turkey

**Keywords:** Aprokam, cefuroxime, cataract, keratoplasty, prophylaxis

## Abstract

**Background:**

Development of endophthalmitis during cataract surgery is one of the most severe complications and it may also result in blindness and globe loss.

**Aims:**

To evaluate the effect of an ophthalmic cefuroxime solution (Aprokam^®^) that is prophylactically used in phacoemulsification surgery performed due to cataract that eventually occurs in patients with or without penetrating keratoplasty.

**Study Design:**

Case-control study.

**Methods:**

In this retrospective study, patients who had undergone penetrating keratoplasty and for whom phacoemulsification surgery was performed due to cataract and an intracameral ophthalmic cefuroxime solution (Aprokam^®^) was administered for prophylaxis were evaluated in comparison with patients with cataract without keratoplasty. The following parameters were evaluated: postoperative anterior chamber reaction, intraocular pressure, best corrected visual acuity, corneal endothelial cell density, and central corneal thickness alterations.

**Results:**

A total of 42 patients (18 males, 24 females) with keratoplasty and 42 patients (17 males, 25 females) without keratoplasty were included in this study. An increase in visual acuity was observed in all the patients after cataract surgery (p<0.001). The mean corneal endothelial cell densitys were determined to be 2437.20±148.5 cells/mm^2^ preoperatively in the study group and 2807.1±152.4 cells/mm^2^ preoperatively in the control group. The mean corneal endothelial cell densitys were determined to be 2103.85±196.5 cells/mm^2^ after the first postoperative month (p<0.001) in the study group and 2755.92±182.7 cells/mm^2^ in the control group (p=0.17). The mean central corneal thickness in the study group were 565.78±11.5 μm preoperatively and 584.65±12.7 μm after the first postoperative month (p<0.001). No difference was observed in the control group in terms of preoperative and postoperative central corneal thickness and endothelial cell density. There was no significant difference between the groups in terms of the intraocular pressure, anterior chamber reaction.

**Conclusion:**

There was no significant effect of the prophylactic intracameral use of ophthalmic cefuroxime solution (Aprokam^®^) on the endothelial cells which was used in patients with keratoplasty for whom cataract surgery was performed.

Cataract surgery is one of the most commonly performed operations throughout the world. The development of endophthalmitis during cataract surgery is a severe complication that may result in blindness and globe loss. The incidence of endophthalmitis has been reported to be between 0.04% and 0.4% (1). Intracameral antibiotic use is one of the most effective methods for the prophylaxis of endophthalmitis during cataract surgery (2). In one study, the European Society of Cataract and Refractive Surgeons reported that the use of intracameral cefuroxime reduces the risk of endophthalmitis by 4.92-fold (2,3). Cefuroxime is among the second-generation cephalosporins, and it inhibits bacterial cell wall synthesis after binding to protein-binding proteins. This effect leads to the impairment of cell wall (peptidoglycan) biosynthesis and, in turn, the lysis and death of bacterial cells (4,5,6,7). Each Aprokam^®^ preparation consists of 50 mg of cefuroxime powder and 9 mg/mL (0.9%) of sodium chlorine for dilution in a 5 mL injection. For usage, 0.1 mL of the prepared solution (1 mg of cefuroxime) is drawn and administered intracamerally (4). Thus, dilution occurs at once, the development of failure of dilution is prevented, and the risk of contamination is reduced, as the dosage is prepared separately for each patient. The cornea is a transparent tissue in which the innermost layer is formed by the endothelial cells. The endothelial cells are hexagonal-shaped, which creates a tightly adhered structure. They have an important function in maintaining the transparent nature of the cornea. Specular microscopy is a noninvasive photographic technique that facilitates the evaluation of the corneal endothelium. This computer-assisted technique can evaluate the shape, size, and number of endothelial cells. For endothelial cell evaluation, specular microscopy is the most commonly used examination method in clinical practice (8). In this study, prophylactic cefuroxime was intracamerally administered in the keratoplastic eyes of patients for whom we performed cataract surgery. Its effect was evaluated in comparison with patients without a history of keratoplasty.

## MATERIALS AND METHODS

This retrospective study was performed at a single center during February 2014-June 2016 in accordance with the Declaration of Helsinki. The study protocol was approved by the local ethics committee (06.01.2017-7911). Patients with or without penetrating keratoplasty were included in the study for whom phacoemulsification surgery was performed due to cataract and prophylactic intracameral cefuroxime solution (Aprokam^®^) was administered. The following parameters were evaluated: postoperative anterior chamber reaction, intraocular pressure (IOP), best corrected visual acuity, corneal endothelial cell density, and central corneal thickness alterations. The IOP was determined using non-contact tonometry, and the central corneal thickness was measured using ultrasonic pachymetry.

### Patient selection

For this study, patients for whom penetrating keratoplasty and cataract surgery were performed due to previous corneal pathologies (stromal dystrophy, n=9; keratoconus, n=12; herpetic stromal scar, n=8; bullous keratopathy, n=13) were selected. Patients without keratoplasty for whom cataract surgery was performed were selected as the control group. All patients in both groups had nuclear cataracts of grade 2-3 or posterior subcapsular cataracts. For all the keratoplasty patients, cataract surgery was performed at least 6 months after the date of penetrating keratoplasty. Patients aged 18 years and older were included. Patients who had glaucoma, uveitis, or diabetes; were using topical prostaglandin analogs, systemic immunosuppressive agents, or anticoagulant drugs; or experienced intraoperative complications (posterior capsule rupture, vitreous loss, elongation of duration of surgery, etc.) were not included in the study. Furthermore, patients who had undergone any type of ocular surgery other than keratoplasty were excluded.

### Surgical technique

**Figure 1:**
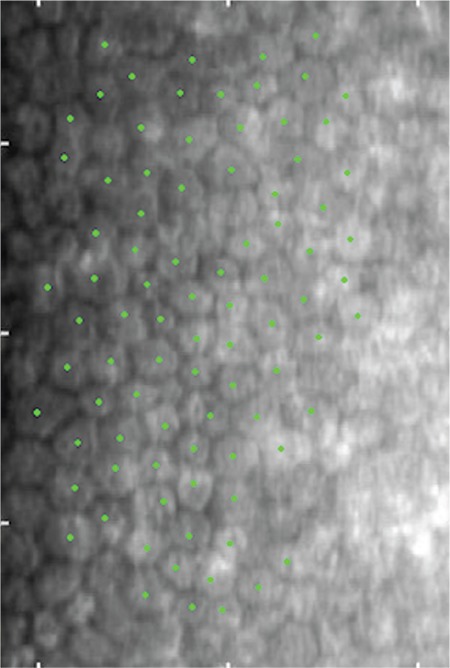
Preoperative specular microscopic image of a cataract patient with keratoplasty. Endothelial cells were counted with the center method.

**Figure 2:**
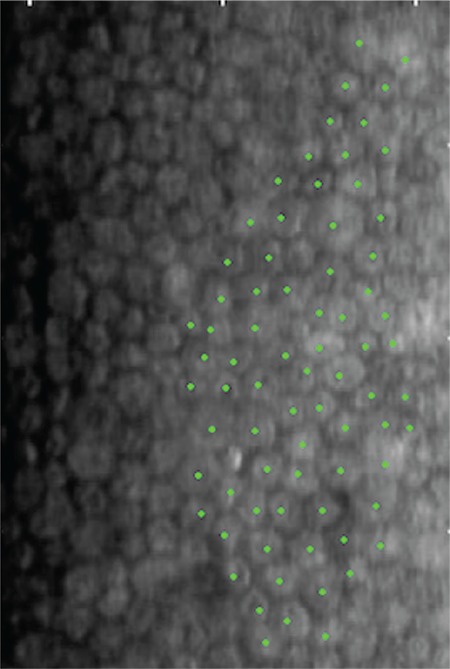
Preoperative specular microscopic image of a cataract patient without keratoplasty. Endothelial cells were counted with the center method.

All surgeries (penetrating keratoplasty and cataract surgery) were performed by the same surgeon. Penetrating keratoplasty was carried out under general anesthesia for all patients, and the diameters of the donor corneas varied between 7.5 and 8.00 mm. The donor cornea was prepared to be 0.25-0.50 mm larger than the recipient bed was. The donor cornea was sutured to the recipient bed using 10-0 nylon sutures with 16 pieces of separated and continuous sutures. Cataract surgery was performed under local anesthesia. The pupil was dilated using 1% cyclopentolate, 1% tropicamide, and 2.5% phenylephrine drops. As a topical anesthetic drop, proparacaine (Alcaine) was used. A major incision of 2.4 mm in size was made in the cataract surgery, and for stabilization of the anterior chamber and endothelial protection, 3% sodium hyaluronate, 4% chondroitin sulfate (Viscoat), and 1% sodium hyaluronate (Provisc) were used during capsulorhexis and intraocular lens (IOL) implantation. An AcrySof SA60AT IOL (Alcon Laboratories Inc., Fort Worth, TX) was implanted. Before beginning the surgery, the lids, eyelash roots, eyebrows, forehead, nose, cheeks, and the temporal orbital region were cleaned with 10% povidone iodine in all patients, followed by a 3 min waiting period. Before surgery, the conjunctival sac was soaked in 30 mL of balanced salt solution after the application of povidone iodine. In the final phase of the surgery, the Aprokam^®^ preparation (including 50 mg of cefuroxime powder and 5 mL of sterile 0.9% solution) was intracamerally administered along with the addition of 0.1 mL of the solution that was prepared through dilution (1 mg of cefuroxime) through the lateral entrance via a 27-gauge cannula. At 2 h postoperatively, the eyes were opened, and topical moxifloxacin and dexamethasone drops were dripped in once every 2 h until the patient fell asleep. From the first postoperative day, the application was carried out six times for a week, and during the following 2 weeks, it was stopped by reducing the medication dose gradually. Specular microscopic examination of the patients was performed by the center method using a non-contact specular microscope (Cellcheck SL, Konan, Japan). Three successful measurements were taken, and blinking was requested after every measurement. The images with the best quality were analyzed. At least 75 cells were signed manually, and the patients’ corneal endothelial cell density value was evaluated using the analysis software (Figure 1, 2). The corneal endothelial cell density and the central corneal thickness of the donor cornea were evaluated preoperatively and after the first postoperative month. An IOP of 10-21 mmHg was accepted as normal, whereas an IOP of >21 mmHg was considered as high. Cells in the anterior chamber were examined under maximal magnification under a slit lamp, which was 2 mm in length and 1 mm in width. They were graded according to the number of cells as follows: trace = 1-5 cells, +1 = 6-15 cells, +2 = 16-25 cells, +3 = 26-50 cells, and +4 = >50 cells.

### Statistical analysis

Data analysis was performed using SPSS software (version 21.0, SPSS, Inc.). Preoperative and postoperative comparisons of the central corneal thickness and corneal endothelial cell density were performed using the paired samples test. The best corrected visual acuity was analyzed using the Wilcoxon Signed-Rank test. A p value less than 0.05 was considered as statistically significant.

Power analysis was performed according to the a priori power analysis approach using the “endothelial cell density” variable as the primary criterion. The minimum required number of subjects was calculated as 26 by accepting the probability of type 1 error (significance level) as 0.05, the power of bivariate tests as 95% (type 2 error as 0.05), and the effect size as 0.75 to compare the mean values of preoperative and postoperative measurements of paired samples. G-power software (version 3.1.9.2) was used for power analysis.

## RESULTS

A total of 42 patients (18 males, 24 females) with keratoplasty and 42 patients (17 males, 25 females) without keratoplasty were included in this study. The mean age of the patients with keratoplasty was 66.16±2.93 years (range: 42-80 years), while that of the control group was 65.86±4.81 years (range: 40-78 years). An increase in visual acuity was observed in all the patients after the cataract surgery. The mean best corrected visual acuities were 0.15±0.09 preoperatively and 0.53±0.16 postoperatively for the study group and 0.16±0.1 preoperatively and 0.9±0.08 postoperatively for the control group; these values were determined to be statistically significant (p<0.001). Postoperatively, in the anterior chamber, the scores of 2+ in 5 eyes, 1+ in 10 eyes, and trace amounts of cells were determined in the study group, while scores of 2+ in 4 eyes, 1+ in 11 eyes, and trace amounts of cells were observed in the control group. In the following days, these cells in the anterior chamber regressed.

The postoperative IOP values for both groups were in the range of 10-20 mmHg, and none of the patients had values >21 mmHg. The mean corneal endothelial cell densities were determined to be 2437.20±148.5 cells/mm^2^ preoperatively in the study group and 2807.1±152.4 cells/mm^2^ preoperatively in the control group. The mean corneal endothelial cell densities were determined to be 2103.85±196.5 cells/mm^2^ after the first postoperative month (p<0.001) in the study group and 2755.92±182.7 cells/mm^2^ in the control group (p=0.17). The central corneal thicknesses in the study group were 565.78±11.5 μm preoperatively and 584.65±12.7 μm after the first postoperative month; an increase of 18.87 μm was determined in the mean central corneal thickness in the first postoperative month (p<0.001). The central corneal thicknesses in the control group were 562.81±12.5 μm preoperatively and 564.72±9.3 μm after the first postoperative month. No difference was observed in the control group in terms of preoperative and postoperative central corneal thicknesses (p=0.43; Table 1, 2). The elective phacoemulsification times were 4.22±1.15 s in the study group and 4.17±1.2 s in the control group (p=0.84).

## DISCUSSION

Cataract surgery is one of the most commonly performed operations globally. The development of an infection during the surgery is one of the most feared complications, and the use of intracameral antibiotics for the prophylaxis of infection has been shown to be effective. In our study, the effect of using an ophthalmic cefuroxime solution (Aprokam^®^) for the prophylaxis of cataract surgery performed in keratoplastic eyes was investigated in comparison with patients without keratoplasty for whom cataract surgery was performed. While an anterior chamber reaction was observed on the first postoperative day, this reaction regressed in the following days. We interpret our observation as being comparable to the temporary reaction that we encounter after the intracameral cefuroxime injection we use during normal phacoemulsification surgery. We believe that this situation does not arise from the toxicity of the drug, but instead, it is a reaction induced by the surgical manipulation. Moreover, in various small-sized studies, ocular toxicity has not been observed with the intracameral administration of the recommended dose (1 mg of cefuroxime) after cataract surgery (9,10). In our study, the control group showed similar results in terms of the anterior chamber reaction.

Increased IOP after cataract surgery has a negative effect on the endothelial cells. Ahmed et al. (11) reported an IOP >28 mmHg in 18% of patients without glaucoma in the early (3-7 h) postoperative period; the IOP decreased to low preoperative levels by 4 days in most of the cases. In another study, different viscoelastic materials were used, and while Healon GV (1.4% sodium hyaluronate) was associated with the largest increase in the postoperative IOP, Viscoat was associated with the lowest increase (12). Consistent with these findings, in all our patients, Viscoat was used, and no increase in the IOP was observed postoperatively. A good cleaning of the viscoelastic material is as important as the properties of the material. Ocular toxicity develops in cataract surgery due to the administration of intracameral cefuroxime at doses higher than the recommended dose. Another cause is the failure of the dilution made during the preparation of the cefuroxime solution (13,14). Delyfer et al. (13) prepared a cefuroxime dose of 40-50 mg, which was administered to six patients; anterior segment inflammation, serous retinal detachment, and macular edema were observed. In contrast, no ocular toxicity was observed in any of the six cataract patients for whom the intracameral cefuroxime dose was determined to be 3 mg, which was administered by dilution (15). Aprokam^®^ is prepared by diluting it at once, and its ready solution is used for dilution. Thus, the risk for the failure of dilution or contamination decreases. In our study, a statistically significant decrease in the number of corneal endothelial cells in the first postoperative month and a significant increase in the corneal thickness were determined in patients with keratoplasty. No change was observed in the control group in terms of corneal endothelial cell density or central corneal thickness. In healthy eyes that have not been exposed to any type of ocular surgery, the number of endothelial cells decreases annually at a rate of 0.6% (16). This rate exhibits an increase after cataract surgery, and it has been reported to be 2.5% in a period of 1-10 years (17). In a study by Shimmura et al. (18), the mean rate of endothelial cell loss at the third month after the cataract surgery in cases with penetrating keratoplasty was reported to be 15.7%±7.3%. In our study, the rates of corneal endothelial cell loss were similar, and the intracameral administration of 1 mg of cefuroxime did not exhibit any toxic effects on the endothelial cell density. Consistent with the previous reports, a 13.7% decrease in the corneal endothelial cell density was observed in our patients with keratoplasty during the postoperative examination after 1 month. No significant difference was observed in the control group in terms of endothelial cell density or central corneal thickness. The short follow-up duration is a limitation of our study, as the corneal endothelial healing process may last until the sixth month postoperatively (19). Further studies may address this issue with a longer follow-up duration.

In conclusion, a significant decrease in the endothelial cell density of the patients with keratoplasty for whom cataract surgery was performed was detected at the first month of postoperative examination. This finding was consistent with the literature. In addition, no change was observed in the control group in terms of endothelial cell density. It was determined that use of an ophthalmic cefuroxime solution (Aprokam^®^) for prophylaxis after cataract surgery in patients with keratoplasty is safe. There was no significant toxic effect of the prophylactic intracameral use of the ophthalmic cefuroxime solution (Aprokam^®^), which was used in patients with or without keratoplasty for whom cataract surgery was performed.

## Figures and Tables

**Table 1:**
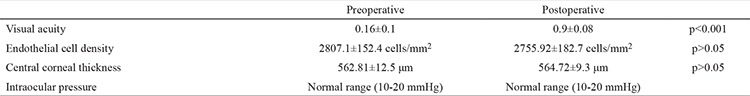
Preoperative and postoperative results of the control group

**Table 2:**
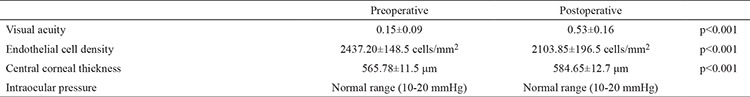
Preoperative and postoperative results of the patients with keratoplasty for whom cataract surgery was performed
